# The Bubonic Plague and Chinese Remedies

**Published:** 1911-03-04

**Authors:** 


### SPECIAL ARTICLES.

### THE BUBONIC PLAGUE AND CHINESE REMEDIES.

(From a Correspondent.)

Now that the plague (in some journals called the Black Death) is raging in Manchuria and North China it is interesting to see how and in what way the Chinese Imperial Government proposes to grapple with this disease.

In Kwangtung and Kwangsi, where it has for the past ten years or more been annually recurrent, very little has been done beyond a rather grudging adoption of some of the foreign methods of dealing with it, and as these were more in the nature of precautions than remedies, it may be truthfully said that plague has usually been suffered to take its course. It is much to be regretted that the researches of foreign physicians, both in India and China, have so far failed to discover any effective cure or preventive of the disease, and Western science has perforce had to be content with the application of mitigating measures in the case of its appearance and disinfection to prevent its spread.

### Chinese Apathy.

The Chinese, on the other hand, seem to know of no remedial measures, and they are in most cases too careless or indifferent to undertake any organised steps to secure disinfection or prevent the disease becoming epidemic. The Imperial Government has now, however, taken alarm, possibly by reason of the outbreak so near to the capital itself, and the dread that, owing to the notoriously insanitary condition of its streets, it may find a great breeding-ground there and become locally a disastrous annual epidemic. Animated no doubt by this all too well-founded apprehension the authorities are summoning the services of all the foreign-trained native doctors available, having obviously no faith in the old-fashioned empirical practitioners on whom they have hitherto depended. It is believed that if foreign doctors were on the spot, their services would now be eagerly sought, as the number of Chinese who have received Western training in medicine is very small. The Hong Kong College of Medicine has turned out quite a number of intelligent young doctors during the past ten years, but these have been scattered, and most of them find employment in South China. The number of medical students will be greatly increased with the establishment of the Hong Kong University, but neither the present nor the immediate future outturn of qualified Chinese doctors from Hong Kong will suffice to meet the present or future needs of China when visited by plague. Foreign assistance is urgently needed, and though hitherto the medical aid given by foreign medical missions has met with very qualified approval by the mass of the people and gratitude from those only who have personally benefited from the skill of the mission doctors, there are not wanting signs of a larger appreciation of Western methods growing up even among the influential foreign hating *literati*.

The plague, whether bubonic or pneumonic, is very fatal among the Chinese, the deaths averaging fully 80 per cent. of the cases. Among Europeans attacked, on the other hand, the percentage is quite small, the reason generally assigned for this being the greater endurance of the Western and the fact that he is better fed and hence has more reserves of strength to draw upon.

### Chinese Prescriptions.

It must not be supposed, however, that the Chinese doctors apply no remedies or make no attempts to treat cases of plague. As a rule its presence is not suspected until the appearance of the bubo, when the native practitioner prescribes a seed of the *Yi, Mi, Coix lachryma* (Job's tears) to be swallowed whole with some boiling water. This is supposed to cause the bubo to break six hours thereafter, when the patient would be pronounced cured of the disease, and it would only be necessary to treat the bubo, which, of course, would remain a sore. This is a prescription for the purpose:—

"Half an oz. snake's cast-off skin; $\frac{1}{10}$ oz. of beehive; $\frac{1}{2}$ oz. clean human hair; $\frac{1}{10}$ oz. black beans; $\frac{1}{2}$ oz. of *ku lien tzu* (*Melia azederach*); and $\frac{1}{2}$ oz. bitter gourd. These ingredients to be ground up very fine and applied externally."

The above prescription was translated by a foreigner from the Chinese and sent to a Shanghai paper, and was subsequently corrected by a Chinese correspondent. I have embodied the corrections in the reproduction, but I doubt very strongly whether the prescription becomes much more efficacious with the additions and corrections of the English-speaking native.

### Some Personal Experiences.

The bubonic plague, of which I saw a good deal in Hong Kong, where it was extremely fatal, develops very rapidly, and the sufferer often mistakes it for a touch of fever until he suddenly falls down and is carried to hospital, where he remains unconscious until death supervenes. I have known cases where office clerks and coolies were taken suddenly when engaged in their ordinary work and hurried off to hospital, never to return. I have seen a chair-coolie fall into the side channel close to his chair, and when picked up found to be already dead. Probably in these cases the virus had been doing its deadly work for days, but the victims had persevered in their avocations, though feeling what they term in that strange jargon "pidgin English," "Hab got little sick."

Medical science has not yet properly or efficiently grappled with this deadly disease, and there is fame to be won by the savant or the doctor who can discover a cure or preventive. The serum treatment, which at one time was highly lauded, has apparently done very little good in cases where it was tried, and hitherto no efficient vaccine has been found. Prophylactic measures, especially sanitary precautions, must still be the main plank of the plague doctor: prevention rather than cure must still be his main dictum.

